# ODiNPred: comprehensive prediction of protein order and disorder

**DOI:** 10.1038/s41598-020-71716-1

**Published:** 2020-09-08

**Authors:** Rupashree Dass, Frans A. A. Mulder, Jakob Toudahl Nielsen

**Affiliations:** 1 Interdisciplinary Nanoscience Center (iNANO), Aarhus University, Gustav Wieds Vej 14, 8000 Aarhus C, Denmark; 2 Department of Chemistry, Aarhus University, Langelandsgade 140, 8000 Aarhus C, Denmark

**Keywords:** Structural biology, NMR spectroscopy, Computational science, Software, Computer science

## Abstract

Structural disorder is widespread in eukaryotic proteins and is vital for their function in diverse biological processes. It is therefore highly desirable to be able to predict the degree of order and disorder from amino acid sequence. It is, however, notoriously difficult to predict the degree of local flexibility within structured domains and the presence and nuances of localized rigidity within intrinsically disordered regions. To identify such instances, we used the CheZOD database, which encompasses accurate, balanced, and continuous-valued quantification of protein (dis)order at amino acid resolution based on NMR chemical shifts. To computationally forecast the spectrum of protein disorder in the most comprehensive manner possible, we constructed the sequence-based protein order/disorder predictor ODiNPred, trained on an expanded version of CheZOD. ODiNPred applies a deep neural network comprising 157 unique sequence features to 1325 protein sequences together with the experimental NMR chemical shift data. Cross-validation for 117 protein sequences shows that ODiNPred better predicts the continuous variation in order along the protein sequence, suggesting that contemporary predictors are limited by the quality of training data. The inclusion of evolutionary features reduces the performance gap between ODiNPred and its peers, but analysis shows that it retains greater accuracy for the more challenging prediction of intermediate disorder.

## Introduction

Intrinsically disordered proteins (IDPs) fail to form a specific stable 3D structure under native conditions. Instead, they are in a statistical equilibrium involving several more or less unfolded conformations dictated by the local amino acid sequence. The structural dynamics and flexibility found in IDPs has been linked to key biological processes involving regulatory and signaling functions^1–3^. This has led to a growing interest in the structural characterization of IDPs^4–6^. Biophysical techniques for characterizing protein structure, such as X-ray crystallography, small angle X-ray scattering^7^, and NMR spectroscopy can be used for characterizing disorder experimentally. However, the experimental characterization of IDPs is time-consuming, laborious, and expensive. To mitigate this problem, a large number of computational methods that aim to predict disorder from sequence have therefore emerged^8,9^.

Contemporary disorder prediction methods are trained on sets of protein sequences with experimentally annotated disorder/order classification. Recently, we discussed the shortcomings of current disorder classification procedures and introduced the use of NMR spectroscopic data as an alternative benchmark^10^. In short, X-ray crystallography is a widely used criterion for judging disorder, where missing electron density is interpreted as disorder. However, the requirement of producing crystals is not commensurate with the observation of disordered residues. Another frequently used source of annotation, containing more cases of disorder, is the community-maintained DisProt database, which provides annotations based on data from various experimental sources. Unfortunately, DisProt has inconsistent annotations due to the heterogeneous composition of techniques, often lacks position-specific information (e.g. annotation derived from CD and sensitivity to proteolytic degradation), and contains false classifications in some cases^11^. As an alternative, Nuclear Magnetic Resonance (NMR) spectroscopy can provide an accurate and residue-specific description of the structure and dynamics of IDPs. For example, the local variation in NMR ensembles has been used to define a disorder classification^12,13^. However, this classification depends also on the local precision of the NMR ensemble, which can vary substantially depending on the amount of available constraints and the protocol used to enforce the constraints and derive the structural ensemble. Furthermore, all available classifiers are binary, ignoring potentially meaningful intermediate disorder^14,15^. The primary NMR observables, the chemical shifts, are measured routinely, are very precise and provide information on the local structure of proteins in solution^16^. Due to their dynamic nature, IDPs exhibit statistically-averaged “random coil” chemical shifts^17^. Conversely, secondary chemical shifts (i.e. the deviation of measured chemical shifts from random coil chemical shifts) indicate formation of structure, and have therefore been used to quantify order/disorder and conformational propensities in IDPs^16,18–25^.

Previously, we introduced the CheZOD Z-score^11^, which is based on secondary chemical shifts, and quantifies the degree of local disorder on a continuous scale. Z-score profiles were derived for a set of 117 carefully selected, representative proteins (herein referred to as the "117" database), revealing a diverse spectrum of disorder. It was demonstrated that the Z-score scale, besides being a reliable measure of disorder, also agrees well with other measures of disorder, such as missing densities in X-ray structures, structural variation in NMR-derived structural ensembles and positional variation in MD trajectories^10^. The CheZOD database was used to benchmark the performance of 26 disorder prediction methods^10^ by assessing the agreement between the estimated probabilities of order and the experimental Z-scores. A modest correlation was found (best method shows an absolute Spearman correlation coefficient of 0.638), and all prediction methods proved inadequate to predict intermediate disorder/order. Furthermore, it was found that the accuracy of disorder prediction methods was limited by the quality of the training data.

We present here ODiNPred; Prediction of protein Order and Disorder by evaluation on NMR data. ODiNPred was trained on a greatly expanded version of the CheZOD database, with experimental continuous-valued disorder Z-scores for 1325 protein sequences (herein referred to as the "1325" database), which spans a comparable number of disordered and ordered residues. ODiNPred uses a deep neural network and 157 residue-specific sequence features to predict a real-valued Z-score of disorder, which can be converted to a probability of disorder. Previously, the “117” database was used to derive a comprehensive and detailed benchmarking of prediction methods^10^. To align with this analysis, ODiNPred was evaluated on the “117” database in a cross-validation setting and was found to outperform 26 recently-tested prediction methods with a Spearman correlation coefficient between observed and predicted Z-scores of 0.649. Prediction accuracy was equal to that of SPOT-disorder, suggesting that this algorithm is the best currently available that is not trained on NMR data. Furthermore, the performance of ODiNPred was assessed on the full 1325 protein sequences in a cross-validation setting, where it demonstrated superior performance. Four biologically relevant examples are provided to illustrate the utility of ODiNPred to comprehensively categorize protein order and disorder. ODiNPred is accessible at https://st-protein.chem.au.dk/odinpred.

## Results and discussion

### Training ODiNPred on a database with balanced order and disorder

**Figure 1:**
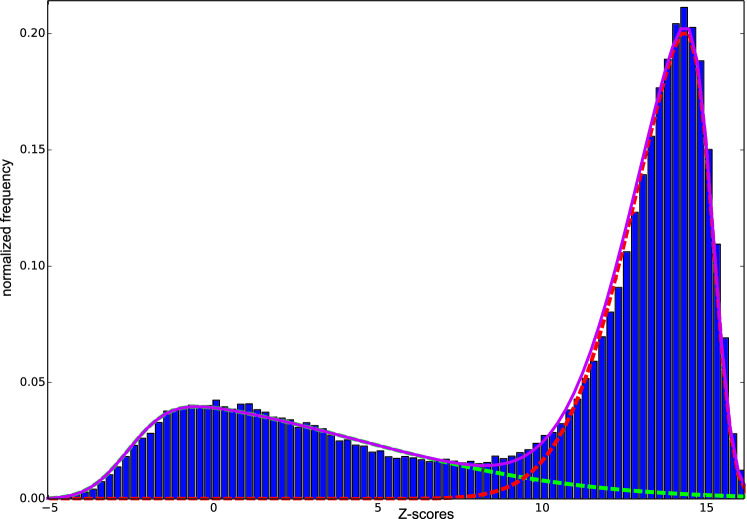
Histogram of all Z-scores in the “1325” CheZOD database used for training ODiNPred. Fits to skew-normal distributions (see “Methods” section) are shown with dashed lines in green and red for disordered, and ordered residues, respectively. A full magenta line indicates the sum of the two distributions.

The CheZOD dataset of 1325 protein sequences and their corresponding Z-scores was used to train ODiNPred. This database was constructed in a way to ensure balanced amounts of disordered and ordered residues (see “Methods” section). A histogram of all pooled Z-scores reveals a bimodal distribution (Fig. 1), as was previously seen before for the "117" database^11^, with Z-scores raging between − 5.0 and 16.15, where the lower end of the scale corresponds to fully disordered residues. The diversity, dynamic range, and balance of the CheZOD database is apparent from the visualization in Supplementary Fig. S1. A threshold value, Z = 8.0, is used to distinguish between disordered and ordered residues^10^. Residues with Z-scores < 3.0 can be considered fully disordered^11^, whereas 3.0 < Z < 8.0 corresponds to cases with fractional formation of local, ordered structure. Conversely, residues with Z > 11.0 correspond to segments of regular secondary structure or structured rigid loops, whereas 8.0 < Z < 11.0 corresponds to flexible loops between ordered segments. To investigate whether the distribution of experimental Z-scores could be interpreted as two broad classes of order and disorder, the distribution was fitted to a weighted sum of skew-normal distributions^26^ as described in “Methods” section. Indeed, a close fit was observed, and this model distribution is henceforth applied here for the statistical inference of disorder probabilities based on experimental Z-scores (see “Methods” section). According to this model distribution, the fraction of disordered residues in our database was 36.3%.

### Disorder propensities for amino acid types

**Figure 2:**
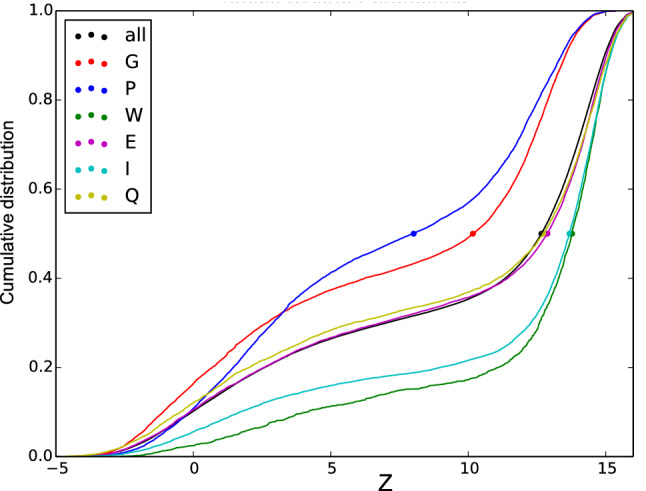
Cumulative distributions functions (CDFs) for Z-scores in the “1325” database used for training ODiNPred. The CDF for all residues combined is shown as a black curve, and CDFs corresponding to specific amino acid types are shown with different colors (see legend). Z-score median values are highlighted by filled circles.

It is well-established that individual amino acid types have different disorder propensities^27–29^. Analysis of the cumulative distribution of Z-scores for individual amino-acid types gives a much more detailed picture along the full scale of disorder and reveals a trend that agrees well with previous findings (Fig. 2); Secondary structure-breaking amino acids, such as glycine, have a larger number of low Z-scores (higher disorder propensity), hydrophobic residues such as tryptophan and isoleucine have the smallest disorder propensities, while hydrophilic/charged side chains give neutral disorder propensities. Figure 2 reinforces that glutamine may be used as a representative for the average behavior of disordered residues^21^. Furthermore, proline displays a very distinct pattern of being disorder-promoting to structured regions, but not to highly disordered segments^30,31^.

### Performance of ODiNPred evaluated by blind prediction of Z-scores

**Figure 3:**
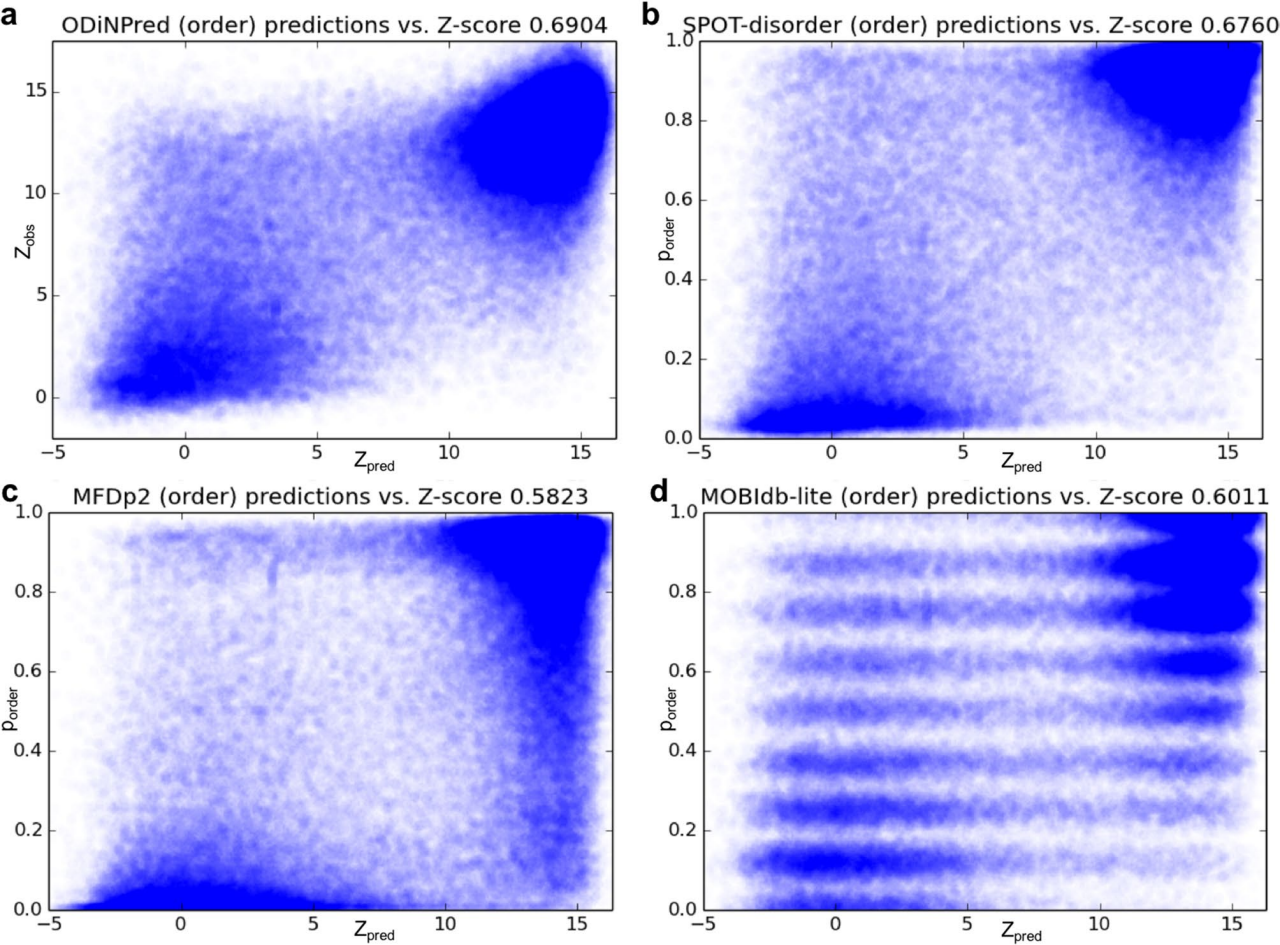
Performance of selected disorder prediction methods on the “1325” cross-validation set. Scatter plots show (**a**) predicted Z-scores (Z_pred_) vs. observed Z-scores (Z_obs_) for ODiNPred for the merged cross-validation sets from CheZOD (see “Methods” section). (**b**–**d**). Probability of order (p_order_ equal to 1 minus the probability of disorder) vs. Z-scores for (**b**) SPOT-disorder, (**c**) MFDp2, and (**d**) MobiDB-lite. Note that MobiDB-lite provides fractions of consensus disorder among eight different fast predictors, hence values are restricted to the rational fractions: 0/8, 1/8, …, 8/8, and white noise with amplitude 0.03 was added to the predictions to allow for better visualization (*nota bene*: the correlation was computed prior to the adding of noise for graphical display).

**Figure 4:**
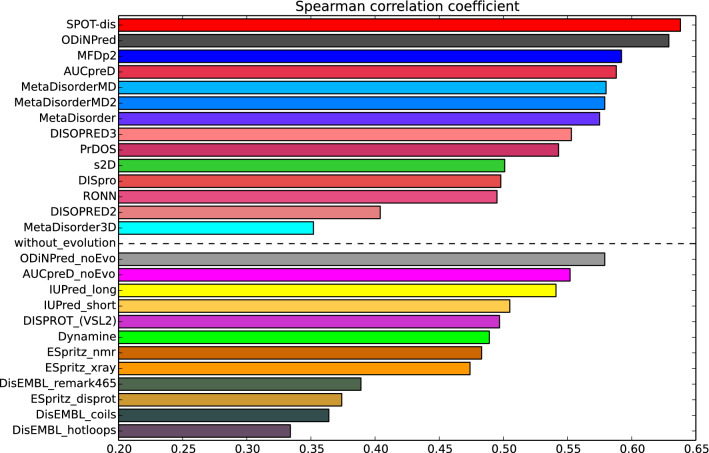
Performance of ODiNPred and other methods on the “117” benchmark set. Spearman rank correlation coefficients are shown as colored bars (see legend to Table 1). A dotted line separates methods that employ evolutional features (top) from those that do not (bottom).

ODiNPred uses a deep neural network to predict continuous-valued disorder Z-scores by training on the "1325" database and used 157 unique sequence input features as described in “Methods” section. ODiNPred was trained in a tenfold cross-validation setting (see “Methods” section) that allows for blind evaluation of predictions for the 1325 sequences (and for any subset of these as discussed below). We note that cross-validation is not biased by training, since, per construction of the database (see “Methods” section), all sequences in each subset contain no more than 50% sequence identity to any of the sequences in the training set, and only include 5.7% identity on average. Figure 3 shows observed versus predicted Z-scores for the 1325 sequences in the CheZOD database. A good agreement was observed when evolutionary features were included (R_Pearson_ = 0.759), and when these were left out (R_Pearson_ = 0.731). Disorder was predicted for the 1325 sequences using three other popular methods, the fast and accurate MobiDB-lite^32^ and the two top-performing methods from the previous benchmark study on the "117" sequences^10^: SPOT-disorder^33^ and MFDp2^34^. Compared to ODiNPred, a weaker correlation is apparent from the scatter plot in Fig. 3. As an alternative, we also evaluated the Spearman rank correlation coefficients (R_Spearman_), which compare the ranking of order probabilities to the ranking of Z-scores, without assuming that these should be linearly dependent. The highest R_Spearman_ was obtained for ODiNPred (Fig. 3 and Table 1). To estimate the performance of our new prediction algorithm, we compared these results with a comprehensive selection of 26 contemporary predictors from a recent benchmark^10^. In this analysis, R_Spearman_ was computed for the “117” database. It should be noted that, although some of the sequences from the “117” database were present in the “1325” database as well, all metrics were carried out in a strict cross-validation setting (see “Methods” section) to ensure proper blind predictions for validation. The values for R_Spearman_ are shown as a bar plot in Fig. 4. It is apparent that ODiNPred and SPOT-disorder stand out as best-performing. Furthermore, when not using evolutionary features (which leads to significant time savings), ODiNPred is significantly more accurate than other methods. We also note that ODiNPred performs noticeably better than several other NMR-based methods such as ESpritz-NMR, *S*2*D*, and Dynamine, where the latter were trained on continuous-valued target data derived from NMR spectroscopy.

**Table 1:** Spearman rank correlation coefficients for the four disorder predictors compared in Fig. 3. In case of ODiNPred the correlation is between observed and predicted Z-scores. In the other cases the correlation is derived between observed Z-scores and estimated probabilities of order.

Method	R_Spearman_
ODiNPred	0.6904
SPOT-disorder	0.6760
MFDp2	0.5823
MobiDB-lite	0.6011

### Performance of ODiNPred on other benchmark data

To follow standard benchmarking procedures for disorder predictors, we also evaluated the performance of ODiNPred on CASP9^35^ and CASP10^36^ datasets using the binary disorder/order classifier provided by CASP for target values. The area under the receiver operating characteristics curve, AUC (see “Methods” section), captures the ability to simultaneously detect disordered residues while also preventing false classification of ordered residues as disordered. The AUC was used in previous CASP evaluations to assess the performance of predictors. ODiNPred was evaluated using the estimated probabilities of disorder, and for CASP9 and CASP10 datasets, we obtained AUC = 0.760 and 0.790, respectively. In this comparison, ODiNPred is not deemed to be among the best predictors (which range between 0.56 and 0.855 for CASP9 and 0.599 to 0.907 for CASP10^36^). It should be noted, however, that in CASP9 and CASP10 disorder is highly under-represented, contributing only 9.2% and 5.9% of the target set, respectively^10^. Such an overwhelming imbalance suggests that the predictors trained on these datasets were trained to recognize features of order and would, consequently, be overly focused on identifying ordered residues correctly. Indeed, it was found that some predictors that are trained on X-ray data overestimate order^10^. The classical AUC for ROC (AUC-ROC) can be optimistic in cases with pronounced class imbalance. In contrast, the precision-recall would not have this optimism bias and will, in principle, be more suited for imbalanced test sets^37^. We, therefore, derived the AUC for the precision-recall curve (AUC-PR) and found an area of 0.365, compared to ranges between 0.193 and 0.603 in the CASP10 evaluation^36^. However, precision-recall analysis mainly focuses on the performance of the classifier of the minority class^37^, which is neither optimal, as predictors should be balanced to accurately recognize both disorder and order. This impedes an objective comparison with CASP. Instead, we argue that the CheZOD database is likely better suited than the CASP targets for assessing the quality of disorder predictions, since it contains balanced order/disorder that matches experimental observations, has more accurate disorder classification by continuous-valued targets for disorder, and has successfully been used to benchmark disorder predictors^10^. To substantiate this point, we derived the Mathews Correlation Coefficient (MCC) and AUC-ROC for the “1325” cross-validation set, using a threshold CheZOD value Z = 8 to demarcate disorder and order. Following this definition, AUC-ROC = 0.914 and MCC = 0.690. These numbers exceed performance indicators for other predictors as well as for various benchmark data sets. For example, for CASP10, the highest MCC is 0.531^36^. This result is in line with our previous study^10^, where also other predictors perform better on the “117” database than on DisProt or CASP X-ray datasets in terms of MCC and AUC-ROC. This reinforces the notion that the NMR-derived Z-score is a reliable and predictable classifier of protein disorder.

### Importance of features

Application of noisy features will lead to over-fitting and will have a negative impact on the performance of neural networks. There appears to be little consensus on how to prune neural networks^38^. Here, Gaussian noise was applied after the first hidden layer to prevent over-fitting. In order to test for over-fitting with a more systematic procedure that provides specific insights, we implemented the permutation importance procedure^39^ to rank features on how important they are in predicting the target scores. This was implemented for each of the 10 validation datasets, shuffling each feature one-by-one (with 5 repetitions) while keeping all other features constant. The optimized neural network construct with all other parameters as described in the “Methods” section was applied. The squared Pearson Correlation (R^2^) between the prediction and the targets were calculated for the cross-validation subset and averaged over the 5 shuffle rounds. A feature was considered important if R^2^ decreased on shuffling it and vice versa. The specific magnitude of the change in R^2^ is not straightforward to interpret (it depends, among other factors, on the total number of features), but the relative magnitude of the change in R^2^ on shuffling was used to rank the features from most to least important (see Figure S2 in the Supporting Information). It was observed that the most important features were those accounting for (i) hydrophobic clusters, (ii) predicted secondary structure, and (iii) evolutionary relationship. These advanced features proved to be more important than the traditionally-applied single amino acid contributions and single univariate features derived from amino-acid specific properties such as isoelectric point. This can be explained by the fact that simple features can be constructed by a linear combination of the amino acid distribution features, whereas the more advanced features will be more orthogonal to the others. Features that account for repeats and linear motifs in a sequence had the least importance. We argue, that this is because these features are sparse (have relatively few non-zero values) and therefore do not contain much information on a statistical basis. However, these might be important for specific cases or for predicting contextual disorder/order as discussed below. In conclusion, all features were found to be important as groups and we, therefore, chose to keep all features.

### Evaluation of ODiNPred on four important examples of disorder in biology

**Figure 5:**
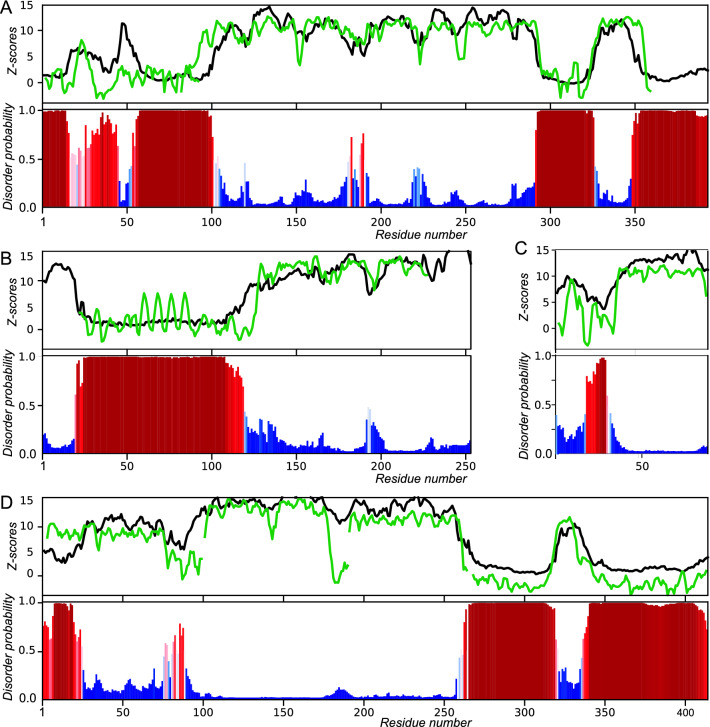
Applications of ODiNPred for disorder prediction. Top panels: Profiles of predicted Z-scores from ODiNPred (black) compared to Z-scores from experimental NMR data (green). Bottom panels: Derived probabilities of disorder estimated by ODiNPred (see “Methods” section) highlighting predicted disordered and ordered residues using red and blue bars, respectively. (**A**) Human oncogene protein p53 (Uniprot P04637, Z-scores from published chemical shifts^41^ and shifts deposited in the BioMagResBank entry 17,760). (**B**) Human Prion Protein (Uniprot P04156. BMRB id 4402^45^). (**C**) DFD *Drosophila* HOX transcription factor (Uniprot P07548, BMRB id 27621^54^) segment. (**D**) TDP-43 (Uniprot Q13148). Z-scores were derived from NMR data from four separately studied domains: (i) N-terminal domain (NTD) residues 3–89 (BMRB id 34081)^62^, (ii) RNA recognition motif 1 (RRM1) residues 91–190 (BMRB id 18765)^63^, (iii) RRM2 domain residues 191–264 (BMRB id 19922)^64^, (iv) Low complexity domain (LCD) residues 268–413 (BMRB id 26823)^59^.

To validate and to illustrate the application of ODiNPred, it was applied to four well-studied proteins that were not part of the “1325” database:

Case 1: The human oncogene protein p53 is involved in numerous protein–protein interactions, which is reflected in its large span of conformations^40^. Recently, we analyzed disorder predictions, disorder annotations and Z-scores for p53, and found that disorder prediction was challenging^10^. With ODiNPred, however, we are now able to demonstrate a close correspondence between predicted Z-scores and those derived from experimental data (Fig. 5A)^41^ (R_Pearson_ = 0.76). Both the disordered terminal regions are correctly identified, as well as the internal disordered region between the two ordered domains. Concurrently, the DNA binding domain (middle part) and the tetramerization domain (res. 325–355) are predicted to be structured (high Z-scores). Furthermore, fluctuations in experimental Z-scores reveal relatively flexible loops (Z-scores between ca. 3 and 10) in the DNA binding domain (middle part), and ODiNPred correctly reproduces these flexible loops, albeit in some cases with slightly larger length or amplitude. It is worth noting that two patches in the otherwise disordered N-terminal domain are predicted to have intermediate order; stretches centered around residue 25 and 50. Indeed, the former region forms a small alpha-helix whereas the latter become structured upon binding of e.g. HMGB1^42^.

Case 2: The human prion protein (hPrP) is associated with fatal transmissible spongiform encephalopathies^43,44^. The structure determined by NMR reveals a folded C-terminal domain and a disordered N-terminus^45^. The C-terminal domain shows high experimental Z-scores and is correctly predicted as structured by ODiNPred (Fig. 5B). Again, it is noticeable that the two larger flexible loops in the C-terminal structured domain are correctly identified by ODiNPred to have increased flexibility. The N-terminal ~ 100 residues have low experimental and predicted Z-scores and, hence, are predicted correctly as disordered by ODiNPred. The quadruple octa-repeat (OR) regions (residues P60-Q91, Fig. 5B) have degenerate chemical shifts for the four repeats and consequently a repeating pattern of Z-scores. Noticeably, the Z-scores for the OR are slightly higher, on average, compared to the other residues in the flanking disordered region. The OR repeat is involved in the misfolding of PrP and constitutes an aggregation locus, influenced by intrinsic flexibility and environmental conditions^46–48^. The OR can adopt stable turn-like structures in the presence of co-factors, such as metal ions and sulfated glycans^49–52^, and these local structures were demonstrated to be transiently present under native conditions. This transient structure is reflected in elevated Z-scores for the OR. Coincidently, ODiNPred predicts slightly higher Z-scores for this segment.

Case 3: The “deformed” (DFD) HOX transcription factor controls the development of the labial and prothorax segments in *Drosophila*^53^. A segment of DFD containing a conserved 60-residue DNA-binding homeodomain and the 30 preceding residues (T337–K426) was studied by NMR spectroscopy and other biophysical characterization techniques^54^. ODiNPred predicts the C-terminal DNA-binding domain to be ordered whereas the 30 N-terminal residues are predicted as mostly disordered (Fig. 5C), in agreement with experiment. More specifically, intermediate Z-scores, 6 < Z < 11, are predicted for residues 2–18 (numbering as in Fig. 5C). Indeed, residues with intermediate experimental Z-scores are part of this segment. Residues 8–11 were demonstrated to be more rigid than the remainder of the disordered N-terminal region by NMR relaxation analysis and MD simulations. This segment with reduced flexibility is specifically recognized by other co-transcription factors^54^.

Case 4: The protein TDP-43^55^ binds to chromosomally integrated trans-activation response element (TAR) DNA and represses HIV-1 transcription^56^. In addition, it is implicated in amyotrophic lateral sclerosis (ALS) and neurodegenerative diseases^57,58^. ODiNPred correctly identifies the folded domains of the N-terminus and the two RNA recognition motifs, having both large experimental and predicted Z-scores (Fig. 5D). ODiNPred correctly predicts the flexible linkers between the isolated domains, which are disordered as defined by Z-scores < 8.0. Furthermore, ODiNPred correctly predicts the C-terminal low complexity domain (LCD) to be disordered, with a segment in the middle of intermediate order, as judged by intermediate observed and predicted Z-scores (residues 320–340). This specific segment has been shown to form a transient α-helix^59,60^ and is involved in liquid–liquid phase separation. The LCD is prone to pathological aggregation, with the α-helical segment mediating tertiary contacts that lead to oligomerization^61^, and mutations in this segment are correlated to ALS. Clearly, ODiNPred is able to accurately pinpoint segments with intermediate Z-scores, which corresponds to sites of important biological function which are implicated in human pathology.

### The full spectrum of order and disorder

ODiNPred extends the repertoire of disorder prediction beyond the categorical and binary disordered and ordered states. We have demonstrated here that ODiNPred can accurately predict flexible parts of otherwise structured proteins as well as segments with transient structure or reduced flexibility within disordered regions (see Fig. 5). Such segments are potentially important for the biological function of many proteins, as the remarkable spatio-temporal heterogeneity of IDPs is closely linked to their interaction promiscuity and multifunctionality^65^. The ensemble of conformations sampled by IDPs constitutes a pre-existing equilibrium in which conformations are available for binding and interaction with ligands or other macromolecules^66^. Furthermore, for structured proteins, the unbound states of flexible loops contain transiently formed conformations that may resemble ligand-bound states^67^. Segments that are partially folded, or have transient residual structure (semi-foldons), as well as segments that can fold dependent on interactions (inducible foldons) are widespread in IDPs and are important for biological function^68^. Protein segments can also be conditionally disordered or transiently disordered depending on the environment and interactions^69^. In addition, a semi-disordered region is prone to aggregation in fully unstructured regions but disposed to local unfolding that exposes the hydrophobic core to aggregation in structured globular proteins. These important “semi-disordered segments” can be identified experimentally as segments with intermediate Z-scores. Since ODiNPred is trained with data covering the full spectrum of disorder, it can predict semi-disorder with confidence, as demonstrated above.

Disordered segments often interact with other proteins serving an important functional role^70^. The IDEAL database annotates a number of protein-binding IDR segments referred to as “protein segments” (ProSs)^71^. Interactions of IDRs have been categorized to occur for three types of segments; LCRs (Low Complexity Regions), SLiMs (Small Linear Motifs), and MoRFs (Molecular Recognition Features)^72^. LCRs are identified by their lower sequence complexity and often by their repetitiveness and are associated with the more generic role of mediating protein liquid–liquid phase separation^73^. SLiMs are distinct short conserved sequences that often mediate the interaction with specific proteins^74^ , and are collected in the ELM database^75^. Features that relate to SLiMs and LCDs were included in the features used by ODiNPred. These features appeared to have limited importance for predicting disorder (see above), which might be due to their limited number of non-zero values. However, these might be important for predicting local variation in disorder and contextual disorder. In contrast to SLiMs and LCDs, MoRFs are a much more general class of longer segments (10–70 residues) that gain some degree of structure upon binding to their targets^76^. MoRFs appear to have some latent propensity to form secondary structure, that could be predicted from sequence features as envisioned in the FELLS analysis^77^ . Other attempts have been made to predicts these segments from more general sequence features with some degree of success^78–82^ . The ANCHOR/ANCHOR2 method differs from other methods, estimating the disordering binding propensity as the product of the disorder probability and the estimated amino acid pair energy gained upon binding^83–86^. For example, for p53 discussed above, ANCHOR/ANCHOR2 assigns a high probability for disordered binding to the N-terminal region (residues 20–60) and the C-terminal region (residues 380–398)^87^, which was also predicted by MoRF_MPM_^80,87^. These regions contain confirmed MoRFs^88–90^ and are annotated as ProSs in the IDEAL database. In agreement, ODiNPred, predicts intermediate probabilities of disorder. This suggests that the MoRF segments have some pre-existing order bias, which is reflected in the dispersion of the average chemical shifts, and thereby gives rise to an elevation of experimental Z-scores, which are mirrored by ODiNPred. This highlights the potency of ODiNPred to predict beyond the order/disorder dichotomy and demonstrates that a comprehensive disorder classification—capturing complex concepts such as intermediate and contextual disorder—is now within reach.

## Conclusions

A new disorder prediction method, ODiNPred, was presented. ODiNPred uses a deep neural network and is trained on experimental NMR-derived Z-scores from 1325 proteins, applying 157 sequence features. When evaluated against a previous benchmark set consisting of 117 proteins, ODiNPred ranked second of 27 prediction methods, with SPOT-disorder showing marginally better performance. When evaluated in a cross-validation setting against the more extensive "1325" database presented herein, ODiNPred displays performance that even surpassed SPOT-disorder. Predictions with ODiNPred can provide key insights, as highlighted with four example cases. ODiNPred can be freely accessed at https://st-protein.chem.au.dk/odinpred.

## Methods

### Datasets

The CheZOD database was expanded to contain 1325 protein sequences with a balanced overall content of disordered residues applying an iterative procedure of adding complementary datasets. As an initial construct, we used the database for training the POTENCI procedure^17^ (containing most of the proteins from the original CheZOD database) keeping the 178 entries having at least 20% residues with Z-scores < 5.0 (f_IDR5_ > 0.2; f_IDR5_ denoting the fraction of residues with Z-scores below 5). Subsequently, this database was expanded with new sequences and their corresponding Z-score profiles derived from chemical shifts deposited in the BMRB database^91^. Entries were only considered if experimental conditions were native, non-denaturing, and non-complexed, as described before^11^. Since the BMRB database has an over-representation of structured proteins, precautions were taken to favor proteins with more disorder in order to construct a balanced database. This was accomplished by adding sequences with progressively more order in separate steps. In each new step, a new candidate set was stripped for sequences with more than 50% sequence identity among themselves and against the previous iteration of the database. Increasingly stricter demands were imposed on the new data to enforce balance. Specifically, in the first iteration, it was required that f_IDR5_ > 0.5 and that the average number of assigned chemical shifts per residue was greater than 2.0 (ACSR > 2.0). In the following iterations, it was required that (i) ACSR > 4.0 for 0.5 > f_IDR5_ > 0.3, (ii) ACSR > 5.0 for 0.3 > f_IDR5_ > 0.2, (iii) ACSR > 6.0 for 0.2 > f_IDR5_ > 0.1, and (iv) ACSR > 6.0 for 0.1 > f_IDR5_ > 0.05 while keeping only the 100 sequences with the largest number of residues. Finally, the new CheZOD database was complemented with sequences from the larger database of structured proteins derived from RefDB^92^, requiring that ACSR > 6 or ACSR > 5.5 and f_IDR5_ > 0.05. It should be noted here that the procedure of requiring fewer ACSR for disordered proteins does not lead to significant statistical bias, since Z-scores are largely unaffected by the number of chemical shifts used to derive them, whereas, in contrast, Z-scores scale approximately linearly with the number of chemical shifts for completely structured residues^11^.

The newly derived CheZOD dataset, containing 1325 protein sequences and their corresponding Z-score values, was then split randomly into 10 disjoint sub-datasets with 132 or 133 entries each. Each of these sub-datasets was utilized for different purposes: (i) a training set used for learning the weights of the neural network, (ii) a testing set used for evaluating the goodness-of-fit of the model after each epoch within the neural network optimization algorithm, and (iii) a validation set used for blind evaluation of the neural network optimized with complementary datasets. Eight sub-datasets were applied for training, whereas a single was used for testing and validation (i.e. 1,060 sequences for training and 132/133 each for testing and validation). The definition of the training/testing/validation sets was varied systematically to define models 1 to 10. In model *n*, sub-dataset *n* and *n* − 1 were used for validation and testing, respectively, whereas the remaining sub-datasets 1, 2, …, *n* − 2, *n* + 1, …, and 10 were used for training. By this procedure, the combined predictions for the validation sets constitute a tenfold cross-validation set. The 10 different models provide slightly different, albeit not independent, predictions for a new protein sequence not present in the "1325" CheZOD database. In such a case, the final ODiNPred Z-score prediction is the average of the predictions of the 10 models. The standard deviation within the 10 different predictions provides an estimate of the precision of the prediction and is further used to estimate the probability of disorder using statistical inference.

Any sequence from the “1325” was always validated in a cross-validation setting. This means that, for a given sequence, only one specific model from the 10 sub-models was used for the prediction. Namely the model, for which the particular sequence was part of the validation subset, and hence not used for neither training nor testing of the neural network. This procedure was also referred to here as blind testing.

### The neural network

Deep neural nets have high capabilities in finding complex relationships between input and output data. ODiNPred uses a feed-forward network^93^ implemented using Tensorflow^94^ with an input layer, five fully connected hidden layers and an output layer with one node and a linear activation function. The input to the network is a matrix of size equal to the length of the protein and the number of features per residue. For the network to learn reliably from the features, each input feature was normalized by its mean and standard deviation. The neural network was set up differently for two cases (i) without and (ii) with evolutionary features. For the case without evolution, the hidden layers contained 40, 10, 25, 40, and 8 neurons, whereas for the case with evolution the hidden layers contained 128, 80, 20, 15, and 10 neurons. In both cases, the response of the first hidden layer was penalized using *L*_*2*_ regularization and the remaining hidden layers used a rectified unit activation function (ReLU). To prevent overfitting, Gaussian noise was applied after the first hidden layer, with a standard deviation of 0.1. The Adam optimization algorithm^95^ was used during training with a learning rate of 0.0001. The mean squared error between the predicted and observed Z-scores was determined for both training and testing datasets after each iteration. The training involved 100 iterations with a batch size of 50 and a standard back-propagation algorithm^96^. The model giving the lowest mean squared error for the testing set was chosen.

### Sequence features

ODiNPred encodes the sequence as a comprehensive set of 157 features derived from sequence attributes such as frequency of amino acids (AA), sequence complexity, secondary structure propensities, and identification of patterns in the sequence such as binding motifs, repeats, and accumulation of identical charges. Known methods such as the Chou–Fasman algorithm^97^ and Tango^98–100^ were also applied for the derivation of some of the sequence features. 27 complementary features accounting for evolutionary relatedness to other sequences were based on sequence alignment profiles generated by BLAST and Clustal^101,102^. ODiNPred predictions can be run optionally with or without calculating and applying these additional features. Most features apply averaging within a sliding window along the sequence. A detailed definition of all features is provided in the “Appendix: Online methods” and Supplementary Table S1.

### Disorder predictions

The distribution of experimental Z-scores was fitted to a weighted sum of two skew normal distributions^26^

$$d\left(x\right)={f}_{D}\psi \left(Z,{\mu }_{D},{\sigma }_{D},{\alpha }_{D}\right)+\left(1-{f}_{D}\right)\psi \left(Z,{\mu }_{O},{\sigma }_{O},{\alpha }_{O}\right)$$

where the skew normal distribution, ψ, is defined in terms of location, scale, and skewness parameters μ, σ, and σ, respectively, as:

$$\psi \left(Z,\mu ,\sigma ,\alpha \right)=2\phi \left({z}_{norm}\right)2\Phi \left(\alpha {z}_{norm}\right), {z}_{norm}=(Z-\mu )/\sigma$$

and ϕ is the standard normal probability density function and Φ is the corresponding cumulative distribution function for the normal distribution. The distribution of the experimental Z-scores agrees well with this model as visualized in Fig. 1 as evidenced by a Hellinger distance of 0.003673^103^. The shape parameters were found by the fitting procedure and the fraction of disordered residues in the training set, *f*_*D*_, were found to be 0.3626.

ODiNPred provides a predicted Z-score, Z_pred_. A probability of disorder, p_D_, is estimated using the fitted shape parameters and a reference fraction of disordered residues, f_Dref_ = 0.333^104^.

$${p}_{D}=\left(\frac{{\pi }_{D}}{{\pi }_{D}+{\pi }_{O}}\right)$$

where

$${\pi }_{S}={\int }_{-\infty }^{\infty }{f}_{D}\psi \left(Z,{\mu }_{S},{\sigma }_{S},{\alpha }_{S}\right)\phi ((Z-{Z}_{pred})/{Z}_{err})dZ$$

with S = D or O denotes the state of order or disorder and again ϕ is the standard normal distribution. The Z-score is predicted using ODiNPred's 10 different cross-validation models and the standard deviation, s_Z_ is extracted from all 10 model predictions and is an indicator of the precision of the prediction. The actual error in the prediction, Z_err_, was compared to the standard deviation for all pooled ODiNPred Z-score predictions for the 1325 sequences. The observations of standard deviations were collected in bins and a clear relationship between precision (average s_Z_ in the bin) and accuracy (average Z_err_) was observed (Supplementary Fig. S3). We used this relationship to estimate the error, Z_err_, on the predicted Z-scores as applied in the equation above.

### Measuring performance

To assess the performance of ODiNPred, the Pearson correlation coefficient is calculated when comparing observed against predicted Z-scores, where a value of 1 indicates a perfect correlation and 0 expresses a complete lack of correlation. The Spearman rank correlation coefficient, describing the agreement with a monotonic relationship was evaluated when comparing disorder probabilities and Z-scores. When assessing the performance against benchmarks with binary classifiers, such as the CASP datasets (for which experimental classification is available), classical binary confusion-matrix parameters, i.e. true positives (TP), false positives (FP), true negatives (TN), and false negatives (FN), were analyzed. The area under a parametric curve is derived using the estimated probabilities of disorder as the parameter to be varied. A receiver operator curve (ROC) is the true positive rate (or recall), TPR = TP/(TP + FN), vs. the false positive rate (false alarms), FPR = FP/(FP + TN), parameterized by the probability threshold, and the corresponding area under this curve (AUC) is an aggregate measure of the quality of the correlation^36^. A perfect classifier would yield AUC = 1, whereas random guessing gives AUC = 0.5.

### Implementation of ODiNPred web application

**Figure 6:**
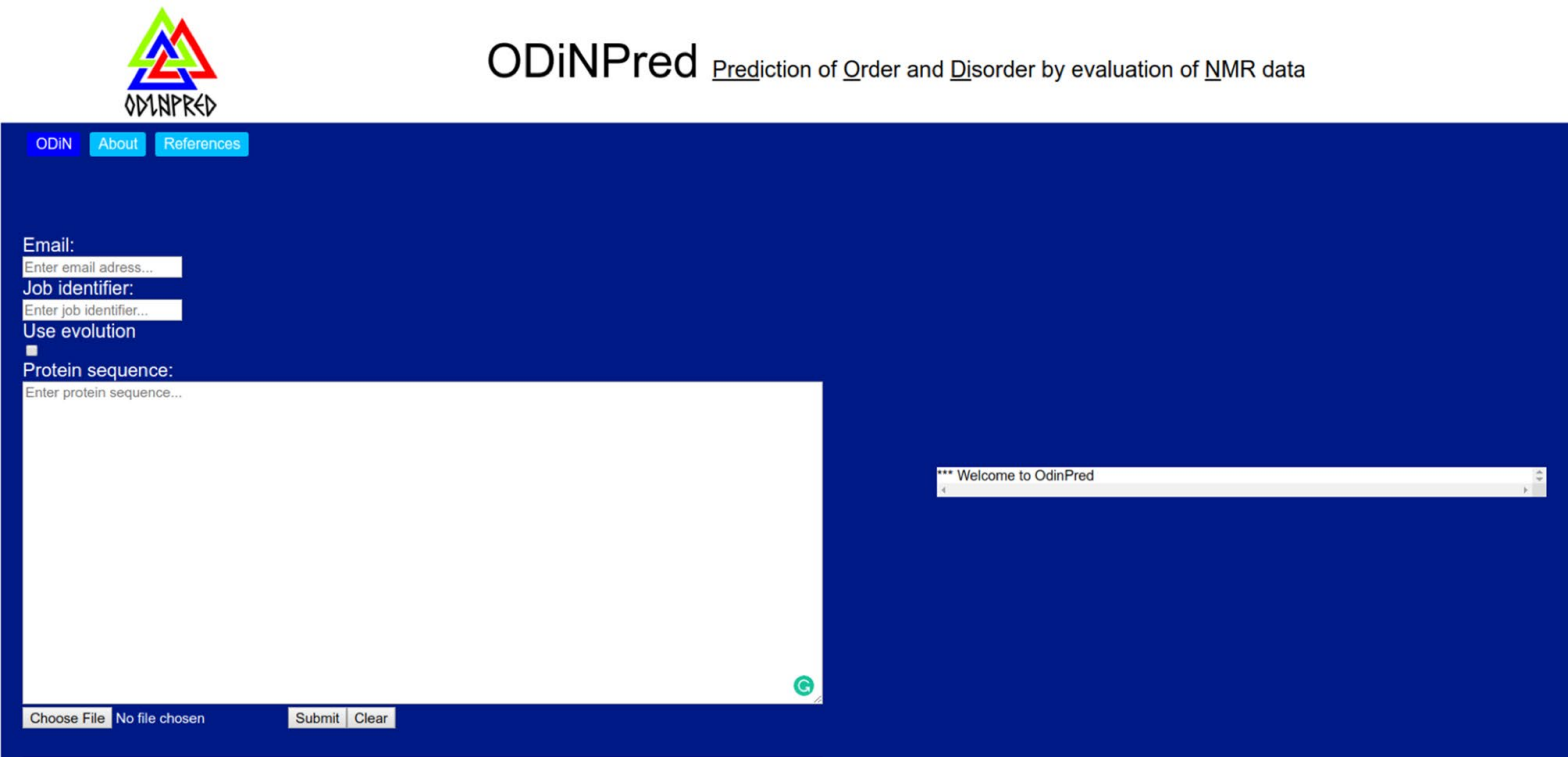
Screenshot of ODiNPred at https://st-protein.chem.au.dk/odinpred.

The ODiNPred web application (run in python) is located at https://st-protein.chem.au.dk/odinpred (Fig. 6). Input data can be uploaded following instructions on the server. ODiNpred can take up to 100 protein sequences as input at a time and predicts their Z-scores and disorder probabilities. It is possible to run predictions with or without evolutionary features included. Running without evolutionary features decreases the run time from approximately one minute to a few seconds per entry on the current server. Including evolutionary features is recommended for predictions of individual sequences, as it produces higher prediction accuracy (see Results). The prediction results are sent by email to the user as a text file and a plot.

### Supplementary material

Supplementary information.

## Data Availability

All sequences from the new 1325 CheZOD database along with corresponding Z-scores are available from: https://github.com/protein-nmr/CheZOD.

## Appendix: Online methods

### Derivation of sequence features

Sequence features were derived from basic procedures and in some cases with the application of external programs or published reimplemented algorithms. Each type of sequence feature is explained in detail below. Some features are based on distributions of amino acids and apply a sliding window to define the amino acid set. For a window size N, (N − 1)/2 dummy residues were added to the ends of the sequences to preserve the length of the sliding windows. Supplementary Table S1 summarizes the length of sliding window and number of features for each type.

#### Amino acid composition

Number of occurrences of each amino acid within sliding window (including dummy end-residue).

#### Sequence entropy

The fraction, f_i_, of each amino acid type was used to derive the Shannon entropy, S^21^, for the local distribution including dummy end-residues.

$${S}^{21}(f)=\sum_{i=1}^{21}{f}_{i}\mathrm{log}({f}_{i})$$

using the definition that the product 0*log(0) is 0.

#### Chou–Fasman secondary structure predictions

The Chou–Fasman algorithm^105^ was used to derive simple estimates of the probability for all secondary structure types (sheet/helix/turn), and averaged using a sliding window (see Supplementary Table S1).

#### Residue interaction entropy

A pairwise interaction energy, E_ij_ was assigned to each residue pair based on the identity of the amino acids^106^. The energies were considered a phenomenological statistical probability, p_ij_, of interaction using the transformation:

$${p}_{ij}=\mathrm{log}(-{E}_{ij}/4.26)$$

This procedure derives a 2d-array of probabilities at each point of the sequence, and Shannon entropy (see above) of this array is the single feature for this type.

#### Flexibility

Window-averaged amino acid specific flexibility index as defined before^107^.

#### Interaction motifs

The sequence was queried for linear motifs listed on https://elm.eu.org/elms^75^ including all types (CLV, DEG, DOC, LIG, MOD, TRG). Each position is assigned a binary value (part of linear motif: 1, not part of: 0). These numbers were used as specific features for each motif type.

#### Net charge

The net charge present in each local sequence segment was calculated using + 1 basic residues lysine and arginine and − 1 for acidic residues glutamate and aspartate.

#### Isoelectric point simple

Window averaged amino acid specific isoelectric point values as defined before^108^.

#### Segment dipoles

The local dipole was calculated for a segment for supposed helical and strand structure using angles of 100° and 170°, respectively, as described by Eisenberg and co-workers^109^, using the amino acid hydrophobicity scale of Kyte and Doolittle^110^.

#### Repeats

Following an iterative procedure, the highest possible number of amino acids, being repeated again, at any position, was identified for each position of the sequence. The probability of the repeat occurring a certain number of times in the full sequence simply by statistical chance was evaluated as:

$$p=\frac{{\mathrm{e}}^{\mathrm{h}}{h}^{k}}{k!}, h={p}_{loc}(N-L+1)$$

where *N* is the length of the sequence, *L* is the length of the repeat, *k* is the number of times the repeats is found (the multiplicities) and p_loc_ is the product of probabilities for each amino acid occurring independently (roughly equal to 1/20^L^). Furthermore, the minimum distance, *d*, in sequence position between consecutive repeats were identified and, in the case of three-times multiplicities of a repeat, the absolute difference, Δ, between two consecutive distances was evaluated (in case of k < 3 the difference is set to 0). Five sequence features were finally generated using sliding window averaging of *L*, *k*, *p*, *d* and Δ.

#### Class-composition/transition

The amino acid types are separate into three disjoints classes, 1, 2, 3 according to different physio-chemical properties according to the definition by protr (see https://cran.r-project.org/web/packages/protr/vignettes/protr.html#4_commonly_used_descriptors, Sect. 4.5 and^111, 112^). For each segment, the fraction of observations of each of the 3 classes defines the class-composition features. At each position, *i*, of the segment, a possible transition between two classes: class(*i*) ≠ class(*i* + 1) was evaluated with four possibilities: no change, change between classes 1 ↔ 2, 1 ↔ 3, or 2 ↔ 3. The fraction of the three different non-trivial transitions defines the class-transition features. Finally, the Shannon entropy among the fractions for the three different transitions was used as the final feature (defined to be 0 in cases of exclusively trivial transitions). This procedure was repeated for seven different definitions of the three-class groupings, yielding a total of 49 features.

#### Secondary structure propensities

The program Tango^98–100^ was used to calculate the local propensity of beta-sheet, turn, helix and poly-proline using standard settings yielding four features.

#### Evolutionary relationships

Evolutionary relationships were derived, ultimately leading to position-specific properties derived from a column of aligned residues generated with the below procedure:

1. Homologous sequences were identified by performing one-by-one alignment with NCBI Blast^113^ initially using the Swissprot database^114^. Sequences, not identical to the query sequence and with an E-value < 0.001 were kept for further analysis. If the accepted number of sequences, N_S_, was less than 10, the full non-redundant database was searched with a similar procedure.
2. Multiple sequence alignment was performed on the above derived set of sequences using ClustalOmega^101, 102^
3. The multiple sequence alignment was organized into a predicted phylogenetic tree using FastTree^115, 116^
4. In case N_S_ > 500, an additional smaller set of sequences was constructed by using Treemer to iteratively remove a single sequence from the phylogenetic tree, being one from the pair of leaves in the tree with the smallest distance. The procedure was continued until N_S_ = 500 and multiple sequence alignment was performed for this smaller set.

Two features were derived from the phylogenetic tree and were set to be constant along the sequence: (i) the average cumulative distance from a sequence leaf to the root of the tree and (ii) the logarithm to fraction between the number of terminal nodes divided by the total number of nodes.

Within each alignment column, the following features were derived (all these features were averaged with a window of 7 subsequently):

i. The fraction of all 20 amino acid types (producing 20 features).
ii. The fraction of gaps.
iii. The fraction of insertions.
iv. The percentage conservation, i.e. the fraction of residues identical to the parent residue.
v. Average blossum62-distance^117^ between column residues and parent residue.
vi. Shannon entropy of the 20-residue distribution within the column.
vii. Jensen-Shannon distance^118^ between the above 20-residue distribution and a reference distribution for proteins in the UniProtKB/Swiss-prot database^119^
viii. Finally, the correlated amino acid mutations feature was calculated. This feature was the most time-consuming scaling with the length of the column and the square of the length of the sequence. Therefore, the smaller, N_S_ < 500, set was used here and the amino acids were grouped into nine groups to effectively use an alphabet of smaller size (ignoring gaps). The ratio between the Shannon entropies, S^9^(f) and S^81^(f), for single alignment columns and the distribution of pairs in combined pairs of columns, respectively, was calculated. The position specific feature, *F*_*i*_, is derived as the average of this ratio for all possible pairs.
  $${F}_{i}=\frac{1}{{N}_{S}}\sum_{j=1}^{{N}_{A}}\frac{{S}^{9}\left({f}_{i}\right){S}^{9}({f}_{j})}{{S}^{81}({f}_{ij})}$$

#### Predicted secondary structure

Secondary structure and solvent accessibility were predicted using the DeepCNF procedure from the RaptorX-property implementation^120^. Following this procedure, probabilities were given for 3-state secondary structure and 8-state secondary structure subtypes as defined by the DSSP algorithm^121^. A number of different features were derived from the probabilities provided by DeepCNF:

i. The 8-state probability (8 position specific features)
ii. The Shannon entropy of the 8-state distribution for each position
iii. The 3-state probability with window averaging of size 15 (3 features)

The states with highest probabilities were analyzed (e.g. if the probability is maximal for helix for a certain position, this is regarded a helical state). Furthermore, we refer to a state with maximal 8-state probability for either “bend” or “no structure” (labels ‘S’ and ‘ ’ by DSSP) as an unstructured state and other states as structured states, and the number of such consecutive unstructured (C_U_) and structured (C_S_) states along the sequence were counted. A number of features were derived using these definitions.

i. log(C_S_)
ii. log(C_U_)

this value was used as a position specific feature for all residues within the specific consecutive stretch. Outside these segments, a value of 0 was used as default.

A 3-state solvent accessibility is provided as well by DeepCNF. Probabilities are provided for states: B (Buried, pACC: 0–10), M (Medium, pACC: 11–40) and E (Exposed, pACC: 41–100), where pACC is the relative solvent accessibility value calculated by DSSP. States were defined as in the above according to maximum probabilities. A number of features were derived:

i. The 3-state probabilities using window averaging of 9 (8 position specific features).
ii. Shannon entropy of the distribution of states within a window of size 9.
iii. The logarithm to the number of consecutive exposed states similar to the above

#### Hydrophobic clusters

Hydrophobic clusters were identified using the HCA algorithm^122^ (Hydrophobic Cluster Analysis). Domains were inferred using the Seg-HCA^123^ procedure implemented in pyHCA^124^. This procedure derives a score, S, and a p-value (p) related to foldability for each domain. The total charge value, C_tot_, and the total absolute number of charges, C_num_, in each predicted domain were identified. A number of features were derived with a fixed value for each residue position in the predicted domain and a default value of 0 for residues outside of the domain:

i. $$\mathrm{log}({L}_{D})$$
ii. $$2\mathrm{log}\left(\frac{0.05}{p}\right)$$
iii. S
iv. $${\mathrm{L}}_{\mathrm{D}}/(10+10{\mathrm{C}}_{\mathrm{tot}})$$
v. $${\mathrm{L}}_{\mathrm{D}}/(10+10{\mathrm{C}}_{\mathrm{num}})$$

Similarly, features were derived for the hydrophobic cluster segments (0 outside segments) using again the total charge and number of charges and here also the number of hydrophobic residues, n_H_, in a segment of length *L*_*S*_

i. $${L}_{S}$$
ii. $$10{n}_{H}/{L}_{S}$$
iii. $${\mathrm{L}}_{\mathrm{S}}/(3+3{\mathrm{C}}_{\mathrm{tot}})$$
iv. $${\mathrm{L}}_{\mathrm{S}}/(3+3{\mathrm{C}}_{\mathrm{num}}).$$

Finally, an array was defined with values set to the difference in primary sequence position between the end and beginning of two consecutive hydrophobic cluster segments. This value was used in the array at all positions from the middle of a segment to the middle of the next segment. The above described array was window averaged with size 13, and the inverse of the array was used for a position specific feature. Two further sequence-specific features were added, in case evolutionary analyses were included. In this scenario, each sequence from the multiple sequence alignment was analyzed by pyHCA to identify the domains with their related p-values. The first feature is the fraction of cases were a domain was identified at a specific sequence position and the second feature is identical to the first except requiring that the p-value was smaller than 0.03 for a domain.

#### Electrostatic potential by statistical mechanics

The electrostatic potential was calculated by adding a dummy charge at each position in the sequence one-by-one and evaluating the electrostatic energy following a statistical mechanics algorithm implemented in pepKalc^125^ using default values for the physical parameters.

#### Length of sequence

Finally, the length, L, of the sequence was included as a constant feature across all positions as: log(min(500,L)).
