# Supplementary material for: ODiNPred: comprehensive prediction of protein order and disorder
Source: Sci Rep. 2020 Sep 8;10:14780. doi: 10.1038/s41598-020-71716-1 (PMC7479119; doi:10.1038/s41598-020-71716-1)
Supplement: Supplementary file 1 — Supplementary information. [file 41598_2020_71716_MOESM1_ESM.docx]

Supplementary Information for:

# ODiNPred: Comprehensive Prediction of Protein Order and Disorder

Rupashree Dass^1^, Frans A. A. Mulder^1,2^, Jakob Toudahl Nielsen^1,2^

^1^Interdisciplinary Nanoscience Center (iNANO), Aarhus University, Gustav Wieds Vej 14, 8000, Aarhus C, Denmark.

^2^Department of Chemistry, Aarhus University, Langelandsgade 140, 8000, Aarhus C, Denmark.

## Supplementary Figures


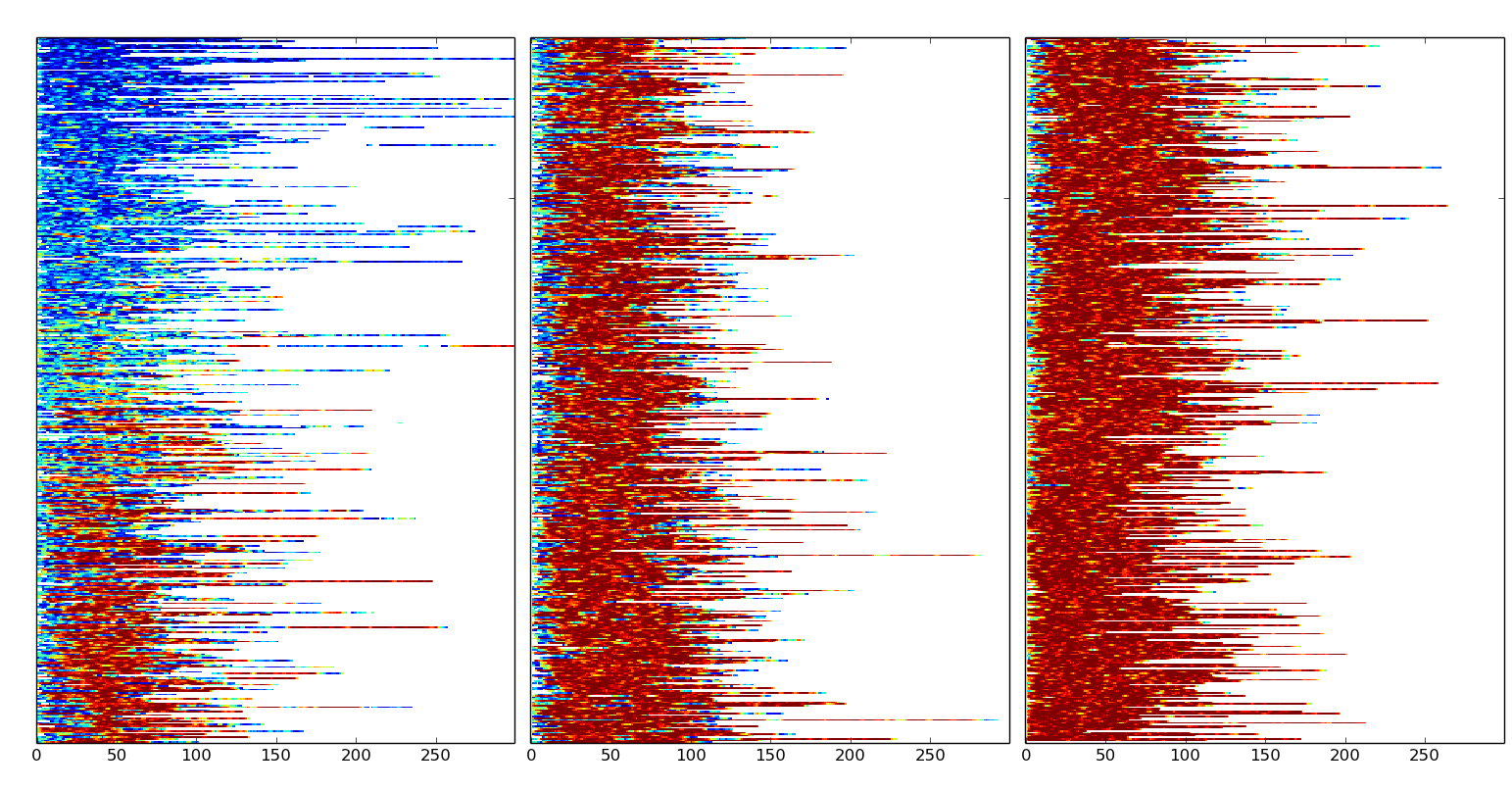
**Figure S1**: Visualization of Z-scores for the "1325" CheZOD database. Each row represents a single protein and Z-scores are visualized using a color ramp from disordered residues (blue-to-green) through to ordered residues (orange-to-red). Entries are ranked from top to bottom according to the average Z-score for each sequence, and shown in one continuous panel that is displayed in three parts from left to right. The x-axis represents sequence numbering.


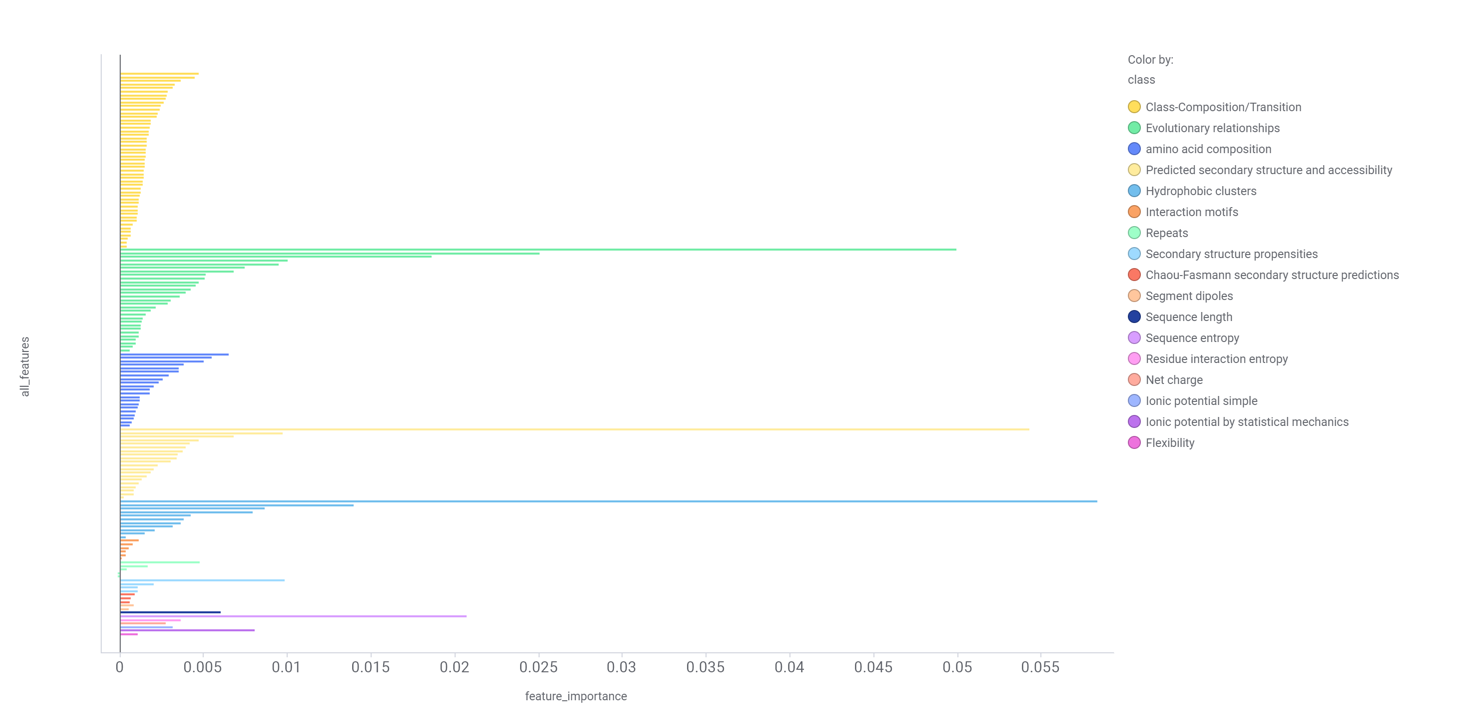


**Figure S2: Feature importance.** The size of the bar is the decrease in squared Pearson correlation coefficient after permutating all values for the corresponding feature (see main text). Higher values correspond to higher importance and vice versa. The specific groups of features, as described in Online Methods and summarized in Table S1, are shown with same colors as given in the legend.

**Figure S3:** Visualization of the correlation between the error in Z-score prediction, Z_err_, and the standard deviation in predicted Z-score, S_Z_, among 10 different ODiNPred models (see Methods main text) shown as a 2D histogram (left) and a scatter plot (right). The average error within the S_Z_ bins is shown by a trend line.

**Table S1: overview of sequence features**

| **Type of feature** | **Sliding window length** | **Number of features** |
| --- | --- | --- |
| Amino acid composition | 25 | 21 |
| Sequence entropy | 51 | 1 |
| Chou-Fasman secondary structure predictions | 25 | 3 |
| Secondary structure propensities | none | 4 |
| Predicted secondary structure and accessibility | different values | 19 |
| Hydrophobic clusters | different values | 12(10^a^) |
| Segment dipoles | 7 | 2 |
| Repeats | 9 | 5 |
| Interaction motifs | none | 6 |
| Class-Composition/Transition | 25 | 49 |
| Net charge | 9 | 1 |
| Flexibility | 7 | 1 |
| Ionic potential simple | 7 | 1 |
| Ionic potential by statistical mechanics | none | 1 |
| Residue interaction entropy | 25 | 1 |
| Sequence length | none | 1 |
| Evolutionary relationships | 7 | 29^b^ |
| **Total** |  | 157(126^a^) |

^a^ Number of features if evolutionary analysis was not included.

^b^ Two versions of the neural network were trained; one with and one without the use of features derived by evolutionary analysis.
